# Entropy and Variability: A Second Opinion by Deep Learning

**DOI:** 10.3390/biom12121740

**Published:** 2022-11-23

**Authors:** Daniel T. Rademaker, Li C. Xue, Peter A. C. ‘t Hoen, Gert Vriend

**Affiliations:** 1 Centre for Molecular and Biomolecular Informatics (CMBI), Radboudumc, 260 Nijmegen, The Netherlands; 2 Baco Institute for Protein Science (BIPS), Mindoro 5201, Philippines

**Keywords:** MSA, entropy, variability, deep learning, amino acids, Philip Bourne, FAIR, bioinformatics

## Abstract

Background: Analysis of the distribution of amino acid types found at equivalent positions in multiple sequence alignments has found applications in human genetics, protein engineering, drug design, protein structure prediction, and many other fields. These analyses tend to revolve around measures of the distribution of the twenty amino acid types found at evolutionary equivalent positions: the columns in multiple sequence alignments. Commonly used measures are variability, average hydrophobicity, or Shannon entropy. One of these techniques, called entropy–variability analysis, as the name already suggests, reduces the distribution of observed residue types in one column to two numbers: the Shannon entropy and the variability as defined by the number of residue types observed. Results: We applied a deep learning, unsupervised feature extraction method to analyse the multiple sequence alignments of all human proteins. An auto-encoder neural architecture was trained on 27,835 multiple sequence alignments for human proteins to obtain the two features that best describe the seven million variability patterns. These two unsupervised learned features strongly resemble entropy and variability, indicating that these are the projections that retain most information when reducing the dimensionality of the information hidden in columns in multiple sequence alignments.

## 1. Introduction

In a recent article [1], Phil Bourne provocatively asked, “Is bioinformatics dead?”. As always with such after-dinner questions, the answer is “Yes and No”, a conclusion that Phil himself already drew implicitly. Phil credits Florian Markowetz for starting the whole discussion [2]. Phil’s article and the three references he cites mention the fourth paradigm—data science—that is to follow the first three: empirical evidence, scientific theory, and computational science [3]. Using Google Scholar, we found many suggestions for the fifth paradigm after data science, e.g., letting computers decide which will be the best experiment to perform next, the mismatch between data-intensive and computer-intensive work, brain–computer integration, network pairing for small sample sizes, or a whole lot more. Zubarev and Pitera’s [4] definition of the fifth paradigm is arguably among the most inclusive and integrative: “cognitive systems seamlessly integrate information from human experts, experimental data, physics-based models, and data-driven models to speed discovery”.

Working with data requires that the data are well-annotated and well-curated, and several articles have been written about the 10 rules for (biological) data storage [5,6]. We agree with Florian Markowetz and Phil Bourne that data science is key to understanding biology, but we also have to deal with the reality that a Google Scholar search for FAIR [7] data results in three million hits, while finding articles that are the result of harvesting multiple FAIR-compliant databases in the bioscience domain is a bit of a challenge. Clearly, a lot of FAIR-related work still needs to be conducted in the worlds of the first three paradigms. The FAIR principles have been applied rigorously to a series of large data collections that are maintained by institutions such as EBI or NCBI, and Phil is one of those whom we should thank for that. Indeed, access to protein, DNA, and RNA sequence data is at the basis of most of today’s understanding of biology and biomedicine. Markowetz [2], for example, asked the question of how one can quantify the genetic heterogeneity that was suggested to be related to the outcome of anticancer therapies. He concluded that “Computational biology excels at distilling huge amounts of complex data into something testable...” and we believe this to be a step towards a fifth paradigm: data-based science (biology) with a human domain expert at the helm. We are starting to see an increasing number of fifth paradigm examples that illustrate the power of the trinity of data science, deep learning, and human domain expertise. It is imperative that artificial intelligence—especially deep learning approaches—will be a tool that is equally as important for this fifth paradigm’s helmsmen as the data science taught in Phil’s school.

The alphafold2 three-dimensional structure prediction algorithm [8], for example, implements human insights and numerous innovations in a deep learning architecture that analyses correlations in multiple sequence alignments (MSAs) to determine which amino acids are in close proximity in three-dimensional space. This follows the philosophy that if it sits together, it evolves together [9,10]. A notable example that demonstrates the power of the data trinity comes from Wang et al., who used deep learning to generate protein scaffolds for user-defined protein functional sites [11]. Mirhoseini et al. demonstrated that the data trinity can even drive AI progress itself by letting a deep learning model create the next generation of Google’s AI accelerators, reducing months of human effort to a few hours [12].

Frameworks have been designed that allow non-AI bioscience domain experts—the helmsmen of the trinity—to combine data with deep learning to answer biomedical questions. An example is DeepRank, a general protein–protein interface analysis framework that outperforms the state-of-the-art algorithms in ranking protein docking models and in classifying biological versus crystallographic interfaces [13].

The alphafold2 experiment lent support to one of the classical ideas in biology that all the data needed to determine the three-dimensional structure of a protein is available in its sequence. Two decades earlier, Laerte Oliveira asked the question of whether the functional role of each amino acid could be extracted from an MSA [14,15]. He showed that this was indeed possible, but in those days, databases were small, computers were slow, and the term ‘deep learning’ still had to be invented. We show here, as an example of the fifth paradigm, that with deep learning, human domain knowledge, and a large set of MSAs, we can reconstruct Laerte’s proposed features to determine the functional properties per amino acid given a protein’s MSA.

The study of protein sequence–structure–function relations has always been a central theme of bioinformatics, and next-generation sequencing has only strengthened this interest. As there are many more protein sequences available than experimentally determined protein structures, multiple sequence alignments (MSAs) dominate this field, as is, for example, illustrated by information systems such as the GPCRDB [16,17] or 3DM [18].

Two principally different philosophies are in vogue to produce MSAs. Classically, MSAs are produced to best represent what happened to the underlying genes during evolution, and the more sequences that can be included, the more information that can be extracted. MSAs that are used in information systems for protein engineering, drug design, and DNA diagnostics, on the other hand, work best if they are centred on one sequence and if all aligned sequences are of similar length. Correlated mutation analysis (CMA) for the purpose of alphafold2-style structure prediction seems to work best using the broader MSAs [9,19,20], while protein engineering and the prediction of residue function normally require one-sequence-centred MSAs.

The information extracted from MSAs is often visualized low-dimensionally using phylogenetic trees or networks of residues that show a high level of mutation correlation. In DNA diagnostics, for example, the degree of conservation at the amino acid residue position where the disease-causing mutation is observed is the single most important factor underlying all analyses [21]; a fully conserved residue position is very important, while a residue found in a maximally variable MSA column is unlikely to be causative for a patient’s disease state. The variability observed in a column in an MSA has been described in many ways, with the Shannon entropy (Σ_i=1,20_ p_i_ × log(p_i_) with p_i_ being the fraction of each of the 20 amino acid types i in column p in an MSA) probably being the most popular. Oliveira et al. [14,15] found large functional differences between columns with similar entropy but different numbers of observed residue types and introduced entropy–variability (EV) plots to combine these two features. These plots proved to be a powerful tool to learn about the function of individual residues.

The use of the EV method to answer biological questions is well documented in the scientific literature. Vollan et al. [22] used the EV approach, for example, to determine the multimeric state of porins. Gaspari et al. [23] used the methods of Oliveira et al. [14,15] to analyse and extend the Pacifastin protease inhibitor family. Wang et al. [24] predicted the early risk of ophthalmopathy in Graves’ disease patients using EV analyses on a patient’s T cell receptor repertoire. Samsonova et al. [25] used the EV method to understand the role of individual residues in the function of olfactory G protein-coupled receptors. Abascal et al. [26] made their model for residue variability among Arthropoda fit the concepts behind the EV method. Bywater [27] used a variant of EV that includes the use of Kolmogorov complexity to extract protein structural features from multiple sequence alignments. These are just a few of the many applications.

**Figure 1:**
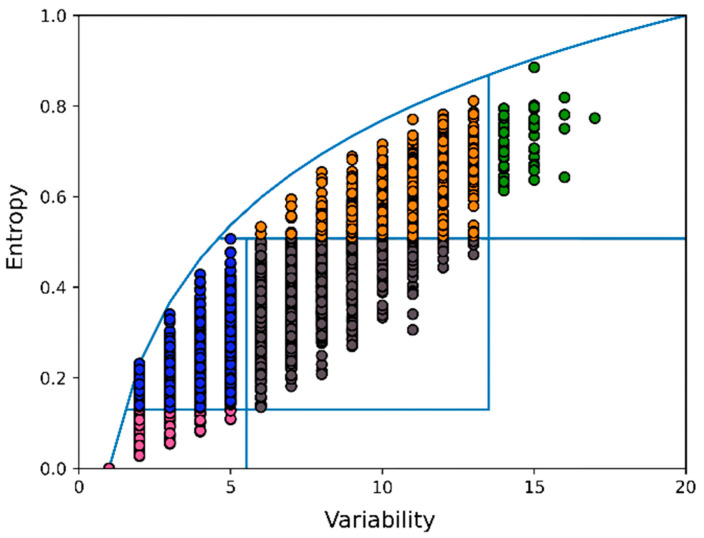
Example EV plot. Colours correspond to functional classes. Each circle represents one column in the MSA, and in this example, thus also one position in the structure. Oliveira et al. divided the EV plot in five areas that—from bottom left to top right—are called Box 11 (pink), 12 (blue), 22 (grey), 23 (orange), and 33 (green). Residue positions in Box 11 were mostly involved in the protein’s main function, while Box 12 residue positions were found in the 3D structure around Box 11 residues. Residue positions in Box 23 were generally associated with modulation (such as ligand-binding residues in receptors, calcium-binding residues in calcium-modulated proteins, etc.). Residue positions in Box 22 tended to be in the 3D structure between residue positions from Box 12 at the one side and residue positions from Box 23 at the other. Residue positions in Box 33, finally, tended to have no discernible function.

EV plots illustrate that residues with either similar entropy or similar variability can still have radically different functions (see Figure 1). Although their method worked nicely for a large series of well-studied proteins, Oliveira et al. could not prove that EV plots were the best way to represent MSAs in two dimensions.

Modern machine learning methods, such as deep learning, have shown to significantly outperform previous methods in many fields [8,28,29]. The power of deep learning lies in the fact that, given enough data, it can fully automatically and unsupervised learn complex features from raw input alone, thereby bypassing the need to create hand-crafted features using the knowledge of a domain expert.

Deep learning models in the biosciences tend to be heavily parameterized, often using large numbers of data types as input and normally using deep learning in a supervised manner for classification purposes. We asked the question of which features would result from a fully unsupervised reduction of the twenty dimensions of an MSA. Using an autoencoder architecture, we stepwise reduced the dimensionality from 20 to 15 to 10 to 5 to 2, while taking great care that at each step the information loss was kept minimal. The features remaining after reduction to two dimensions resemble entropy and variability remarkably well.

## 2. Materials and Methods

Multiple sequence alignments were extracted from the human genome HSSP files [30,31]. This dataset was filtered to remove individual columns where the 20 canonical amino acids contributed for less than 75%. The remaining 7,033,530 columns were each converted to a vector **p** of twenty elements p_i_ that are the fraction of the twenty amino acids i in that column. The elements p_i_ were sorted from high to low.

We combined elements from several well-known techniques [32,33,34] into an autoencoder that is optimal for MSA variability signal reduction. This autoencoder consisted of an encoder with layers of size 20-15-10-5-2 and a symmetric decoder [34]. The input to and the output from the autoencoder are the 20-dimensional vector of the relative frequency for each amino acid; the output vector is the best reconstruction possible of the input vector after passage through the 2-dimensional bottleneck. The network does not make use of tied weights. Batch normalization with parameters [33] was used for all hidden layers. The sigmoid function was applied after batch normalization to all units. Due to the small bottleneck of two neurons, the training procedure consisted of a greedy layerwise pretraining finished with fine-tuning [34]. Parameter optimization was performed via stochastic gradient descent using ADAM [35] with a learning rate of 10^−3^ and a batch size of 128 (i.e., 128 column vectors of p). The design of this autoencoder allowed all training steps to use the fast binary cross-entropy loss function. The binary cross-entropy function (not to be confused with the sequence entropy in columns in an MSA) measures the difference between the 20-dimensional input vector and its reconstruction.

The entropy values in neural plots (neuron1 with respect to neuron2) are normalized to the maximum entropy per variability.

The code was written in Python using the PyTorch library [36]. The resulting autoencoder software is available from GitHub: https://github.com/cmbi/EntVar/, accessed on 1 July 2022.

## 3. Results

**Figure 2:**
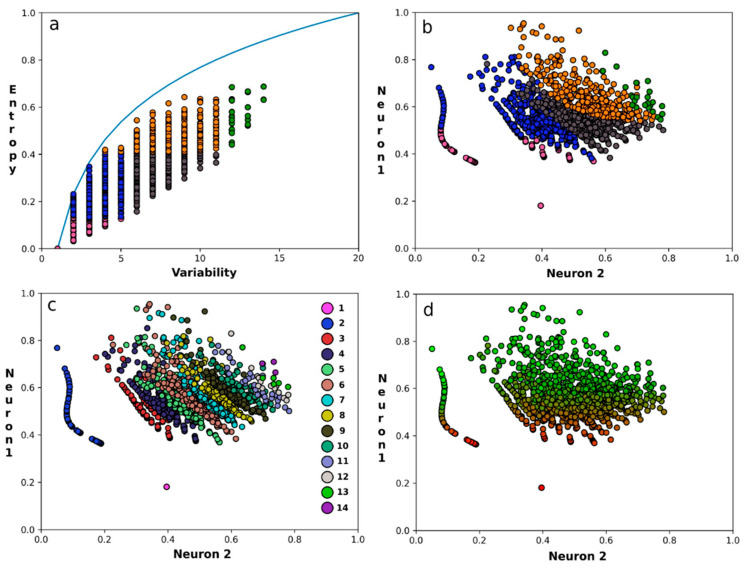
Variability reduction of an MSA. Each circle represents one column from the SPG11 [37] MSA. (**a**) The EV plot according to Oliveira et al. using today’s MSA (colours corresponding to functional classes as in Figure 1). (**b**) Neural residue plot in which each residue is coloured as in A. (**c**) The same as B but coloured by variability. The column of circles at the right-hand side indicates the colour used for the variability values from 1 till 14. (**d**) The same as B, but coloured by relative entropy on a gliding scale from red to green. Neuron 1 and neuron 2 are the two elements of the 2-dimensional bottleneck vector of the autoencoder.

Figure 2a is the classical EV plot for the SPG11 protein. Figure 2b is the neural representation for this same protein, i.e., each column from the SPG11 MSA is now represented in the two dimensions according to the autoencoder’s data reduction. In Figure 2b, each residue is coloured as in Figure 2a. Figure 2c,d are the same as Figure 2b, but coloured by the variability and the entropy, respectively. It is remarkable how well residues of the same colour cluster in the three neural plots. Oliveira et al. analysed well-studied protein families and mapped experimentally determined residue functions on the EV plots. They then drew boundaries between areas where certain types of function were predominantly observed. These boundaries were somewhat arbitrary, and their optimal location depended, for example, on the number of sequences aligned, the average pairwise sequence identity between the aligned sequences, and the function of the protein family. Oliveira et al. realized that the functional classes are not very sharply divided over the EV plot and that it would be better to see the boundaries as guidelines. The mapping of the EV plot colours on the three neural plots supports this latter idea. The neural plots show a clear gradient when going from low to high entropy or variability. The only exceptions are columns with variability 1 or 2, which are separate groups at the bottom left of the neural plots. Since neural networks operate in continuous space, the discrete character of variability is expected to be blurred out in the plot. Columns with variability 1 or 2 are found at distinct locations, while columns with high variability and high entropy tend to not be separated well. In Figure 2d, entropy values are normalized to the maximal value attainable at each of the twenty variability values. When the entropy values are not normalized, the colour gradient in neural plot 2d does not run from bottom to top, but from bottom left to top right.

## 4. Discussion and Conclusions

Oliveira et al. had to read nearly a thousand articles to obtain the data needed to functionally classify residue positions in five well-studied protein families. Their EV plots were an attempt to map residue functions on a human readable representation. We used an autoencoder that completely unsupervised, and without the need to spend years of human effort on feature creation, to obtain essentially the same results.

We used an autoencoder with layers 20-15-10-5-2. Alternate layer schemes such as 20-16-8-4-2, 20-19-18 … 4-3-2, 20-64-32-16-8-4-2, etc., all produced highly similar results.

A reduction to three rather than two dimensions resulted in a three-dimensional distribution of MSA columns (and thus residue positions in the protein’s structure) that we could not relate to anything biologically meaningful. This is partly caused by the fact that there is no literature available in which variability patterns are reduced to three features by either supervised or unsupervised methods. The three features were not entropy, variability, and a third term. Entropy and variability mapped seemingly randomly on the three-dimensional plot.

**Figure 3:**
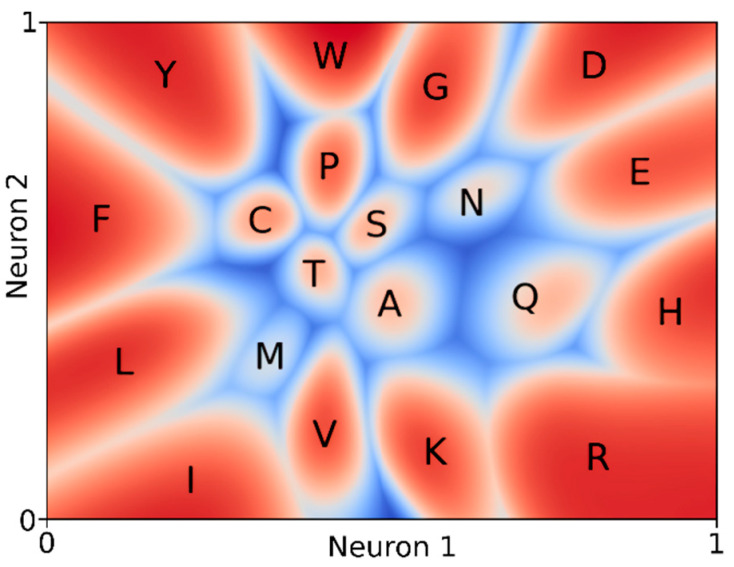
Autoencoder results for the MSA column reduction with fixed order of the twenty amino acid types over the column. These results are beyond the scope of this article but will be discussed extensively on the associated website: https://swift.cmbi.umcn.nl/gv/EV/index.html, accessed on 1 July 2022. Neuron 1 and neuron 2 are the two elements of the 2-dimensional bottleneck vector of the autoencoder.

The 7,033,530 columns were all sorted with the highest residue frequency first to ensure that the autoencoder analysed variability patterns. When the vectors were not sorted so that the twenty elements p_i_ always represented the frequencies of Ala, Cys, Asp, Glu, etc., the two dimensions represented the amino acid types, their characteristics, and their mutabilities in ways that are not surprising to bioinformaticians from Phil’s generation. These results are shown in Figure 3.

Figure 2 and Figure 3 illustrate that the autoencoder software can represent variability patterns in MSAs in two dimensions in ways that correspond well to human knowledge about amino acids and protein sequence–structure–function relations. However, the autoencoder remains a black box. It is impossible to determine how it obtained its results. For example, when the dimensionality of the data gets reduced from twenty to three, no discernible patterns emerged, but with two dimensions the classical EV plot emerges. In Figure 3 we observe that residues with similar biophysical characteristics land close to each other. As these biophysical characteristics are not one single continuum, Figure 3 principally must contain exceptions. Indeed, we observe that the largest residue, tryptophan (W), lies adjacent to the smallest one, glycine (G). Other than the knowledge that hydrophobicity is the most important parameter when comparing amino acid types (and thus that hydrophobicity is ‘more important’ than residue size) we cannot learn from the autoencoder why this is true. Therefore, even though the autoencoder beautifully describes the information in the data, a human expert must still place this information in the wider context of our knowledge, confirming the need for a domain expert in our fifth-paradigm bioinformatics trinity.

## Data Availability

HSSP database downloading instructions can be found at: https://swift.cmbi.umcn.nl/gv/hssp/, accessed on 1 July 2022.
